# Quantifying social distance using deep learning-based video analysis: results from the BTBR mouse model of autism

**DOI:** 10.3389/fnbeh.2025.1602205

**Published:** 2025-06-20

**Authors:** Tausif Khan, Kostiantyn Cherkas, Nikolas A. Francis

**Affiliations:** ^1^Department of Biology, University of Maryland, College Park, MD, United States; ^2^Program in Applied Machine Learning, University of Maryland, College Park, MD, United States; ^3^Brain and Behavior Institute, University of Maryland, College Park, MD, United States

**Keywords:** autism, mice, BTBR, CBA, social distance, DeepLabCut

## Abstract

Autism spectrum disorder (ASD) is characterized by challenges in social communication, difficulties in understanding social cues, a tendency to perform repetitive behaviors, and restricted interests. BTBR T^+^ Itpr3^tf^/J (BTBR) mice exhibit ASD-like behavior and are often used to study the biological basis of ASD. Social behavior in BTBR mice is typically scored manually by experimenters, which limits the precision and accuracy of behavioral quantification. Recent advancements in deep learning-based tools for machine vision, such as DeepLabCut (DLC), enable automated tracking of individual mice housed in social groups. Here, we used DLC to measure locomotion and social distance in pairs of familiar mice. We quantified social distance by finding the Euclidean distance between pairs of tracked mice. BTBR mice showed hyperlocomotion and greater social distance than CBA control mice. BTBR social distance was consistently greater than CBA control mice across the duration of a 60-min experiment. Despite exhibiting greater social distance, BTBR mice showed comparable socio-spatial arrangements of heads, bodies, and tails compared to CBA control mice. We also found that age, sex, and body size may affect social distance. Our findings demonstrate that DeepLabCut facilitates the quantification of social distance in BTBR mice, providing a complementary tool for existing behavioral assays.

## Introduction

Autism spectrum disorder (ASD) is characterized by challenges in social communication, difficulties in understanding social cues, a tendency to perform repetitive behaviors, and restricted interests (Bejerot et al., 2014; Li et al., 2023; Chevallier et al., 2012; Dawson et al., 2005; Kogan et al., 2018; Maenner et al., 2023). For example, individuals with ASD often struggle with recognizing facial expressions, maintaining eye contact, and interpreting emotions (Chevallier et al., 2012; Dawson et al., 2005; Hollocks et al., 2019). Difficulty with social interaction may trigger anxiety in some individuals with ASD, leading to behavioral withdrawal from social interactions due to fear of social contexts or a reduced sensitivity to the positive aspects of social engagement (Bejerot et al., 2014; Li et al., 2023). This pattern of social avoidance may be related to heightened levels of depressive symptoms associated with ASD (Hollocks et al., 2019; Hossain et al., 2020), since greater social support is associated with a lower risk of developing depression (Choi et al., 2023; Choi et al., 2020; Teo et al., 2013). Given the importance of social interaction in health and well-being (Lamu and Olsen, 2016), a primary goal of pre-clinical research on ASD is to understand the genetic and neurobiological basis of social behavior.

The BTBR T^+^ Itpr3^tf^/J mouse strain (BTBR) is commonly used to study ASD because BTBR mice have behavioral profiles that resemble symptoms of ASD (McFarlane et al., 2008; Meyza and Blanchard, 2017), unlike C57BL/6 mice commonly used in social research. For example, when paired with an unfamiliar “stranger” mouse, BTBR mice tend to avoid face-to-face and face-to-body interactions (Ellegood et al., 2013; Scattoni et al., 2013; Yang et al., 2012), which are thought to reflect an avoidance of eye-gaze (Defensor et al., 2011). In general, BTBR mice spend less time with the stranger mouse (Avolio et al., 2024; Meyza et al., 2015), and the avoidance behavior is not thought to arise from aversive odor cues, but rather from the behavioral aspect of social interaction (Ryan et al., 2019). BTBR social interactions with familiar cagemates also show a decreased frequency of both social approach (Pobbe et al., 2010) and following behavior (Winiarski et al., 2022). While it is clear that BTBR mice avoid social interaction, the spatial values that quantify social boundaries between mice have not been established.

It is thought that ASD-like behavior in BTBR mice arises from many genetic mutations and neurobiological factors in regions of the brain associated with social behavior (Meyza and Blanchard, 2017). For example, social experience in BTBR mice enhances c-Fos responses in the periaqueductal gray, a brain region associated with defensiveness. BTBR mice also show low levels of c-Fos responses associated with serotonergic signaling in the amygdala (Higuchi et al., 2023), as well as low GABA levels and high glutamate levels in the amygdala (Bove et al., 2024), frontal cortex (Bove et al., 2024), and auditory cortex (Tang et al., 2024). Accordingly, treating BTBR mice with propofol (a positive allosteric GABAergic modulator) (Cai et al., 2017), or with a selective serotonin reuptake inhibitor (Cai et al., 2019; Chadman, 2011), may rescue BTBR social behavior. A single-nucleotide polymorphism in BTBR mice leads to the deletion of the DRAXIN gene, which causes corpus callosum dysgenesis (Morcom et al., 2021; Dodero et al., 2013; Fenlon et al., 2015; Martin et al., 2021; Miller et al., 2013). Corpus callosum dysgenesis is thought to occur more frequent in individuals with ASD (Alexander et al., 2007; Frazier et al., 2012; Frazier and Hardan, 2009; Travers et al., 2015). Importantly, the brains of BTBR mice show altered resting state functional connectivity (Sforazzini et al., 2016), which is also observed in people with ASD (Rasero et al., 2023; Supekar et al., 2013; Just et al., 2012). Thus, BTBR mice are a valuable model for ASD because their social behaviors, genetics, and neurobiology parallel many core aspects of ASD in humans.

To investigate social behavior in BTBR mice, we used DeepLabCut (DLC) (Lauer et al., 2022; Mathis et al., 2018), an open-source video analysis tool that leverages machine learning to accurately track and label key body parts in multi-animal scenarios. DLC facilitates precise quantification of the spatial units (mm) that define social distance. We used DLC to study social behavior in pairs of familiar cagemates. We compared locomotion and social distance between pairs of BTBR versus pairs of CBA/CaJ (CBA) control mice, expecting that BTBR mice would show greater social distance during experiments. We selected CBA mice in part to evaluate the generalizability of DLC-based tracking across fur colors, as BTBR and CBA mice have dark and light coats, respectively. While C57BL/6 mice have been commonly used as a control strain, BTBR and C57BL/6 mice have distinct genetic backgrounds. Thus, it is important to compare BTBR mice also with alternative genetically distinct control strains, including CBA mice, to contextualize the specificity of BTBR social behavior.

We found that DLC was accurate in tracking both BTBR and CBA mice. BTBR mice displayed hyperlocomotion and remained farther apart, i.e., had a greater social distance, than CBA mice. Several phenotypic factors including age, sex, and body size may contribute to social distancing in mice.

## Materials and methods

### Animals

All procedures were approved by the University of Maryland Institutional Animal Care and Use Committee. We used 19 BTBR T^+^ Itpr3^tf^/J mice (BTBR; 9 females, 10 males; The Jackson Laboratory; stock #000654) and 12 CBA/CaJ mice (CBA; 7 female, 5 male; The Jackson Laboratory; stock #000654), 2–8 months old. Mice were housed under a reversed 12 h-light/12 h-dark light cycle.

### Videoing mouse behavior

**Figure 1:**
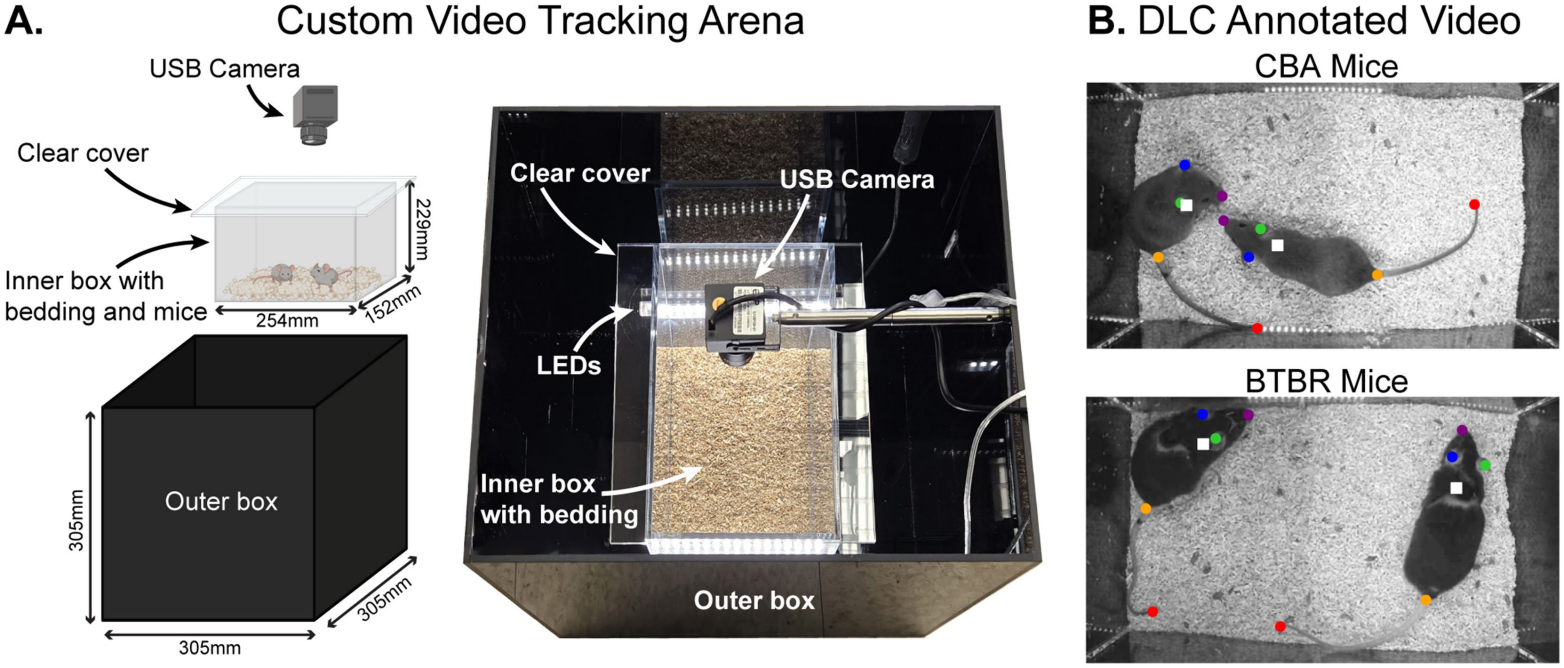
Automated tracking of BTBR T^+^ Itpr3^tf^/J (BTBR) and CBA/CaJ (CBA) mouse behavior using a custom video tracking arena and DeepLabCut (DLC). **(A)** Custom video tracking arena. Pairs of mice were placed in a clear plastic inner box with bedding and a removable clear plastic cover. The inner box was placed within a lidless black plastic outer box. A USB camera was mounted onto the inside of the outer box, facing downward into the inner box to video the mice. **(B)** Example annotated mouse video. Pairs of mice in a cage were videoed for 1 h using the top-view camera. We used DLC to generate labeled body parts on each mouse (colored dots), and we calculated a frame-by-frame centroid of the body parts to yield a single point that tracked each mouse individually (white squares).

We studied mouse behavior using a custom-built video tracking arena (Figure 1A). Pairs of mice were placed in a clear plastic inner box with bedding and a removable clear plastic cover. The inner box was placed within a lidless black plastic outer box with LEDs on the inner walls. A USB camera was mounted onto the inside of the outer box, facing downward into the inner box to video the mice. The open top of the outer box allowed low-level room light into the arena, necessary for videoing behavior. For each video, 2 mice of the same sex and strain (i.e., 2 BTBR or 2 CBA mice) were placed in the arena. A total of 31 pairs of mice were recorded (19 pairs of BTBR mice and 12 pairs of CBA mice). Each pair of mice were littermates from the same home cage. To maximize the number of videoed social groups, pairwise combinations of mice from a given home cage were used. Each mouse was videoed only once in a given day, and in 1–3 videos across days. Behavior was recorded at 30 frames per second (fps) for 60 min. After each recording session, all videos were converted to grayscale and segmented into a series of 120 30 s videos for subsequent analysis.

### Determining the size of a mouse

To find the area occupied by each mouse in the video arena, we first found a representative frame in each video in which the mouse assumed a pose that was common across all mice in our experiments. Using custom Python scripts, we manually drew a polygon around the perimeter of each mouse’s body. We then computed the area (mm^2^) of the polygon to find the size of each mouse.

### Tracking social behavior in mice

We used DeepLabCut (DLC) (Lauer et al., 2022; Mathis et al., 2018) to track the location of individual mice in each video and to analyze social behaviors. To train the DLC model, we manually labeled five key body parts for each mouse: the nose, right ear, left ear, tail base, and tail end (Figure 1B) across a total of 3,485 frames. Of these, 95% were used for training and 5% were held out for testing. For each mouse in a recorded pair of mice, we first labeled body parts across 20 frames from 20 different videos, distributed throughout a given experiment. A subset of frames was then relabeled to enhance DLC tracking accuracy.

Training was conducted using a dlcrnet_ms5 based neural network architecture and the Adam optimizer, with a batch size of 8 and a multistep learning rate schedule to simultaneously track both mice in a recording (Lauer et al., 2022). The learning rate was set to 0.0001 for the first 7,500 iterations, then reduced to 0.00005 until 12,000 iterations, then further reduced to 0.00001 for the remaining training steps for up to a maximum of 200,000 iterations. The model’s performance was evaluated on the held-out test set, yielding a mean error of: 8.92 pixels for test frames, and 2.36 pixels for training frames (image size was 480 × 270 pixels). Using the known arena scale (0.68 mm/pixel), these errors correspond to approximately 6.07 and 1.60 mm reflecting the spatial accuracy of the tracking system. A p-cutoff of 0.6 was applied to filter low-confidence predictions, ensuring that only reliable body part estimates were used in subsequent analyses. This network was used by DLC to automatically label the 5 body parts for each mouse throughout each entire video. CSV files containing the spatial coordinates of each labeled body part were generated by DLC and then imported to Python or MATLAB for a given analysis.

To track social distance between pairs of mice M1 and M2 in a video, we first computed the centroid of each mouse based on the average position of all its labeled points. We then found the Euclidean distance between centroids of M1-M2 in each 30 s video within the 60-min video session. We also quantified locomotion as the spatial displacement of a single tracked centroid per second. Displacements for a given mouse were calculated by taking the Euclidean distance between its centroid locations in consecutive frames.

To quantify the socio-spatial arrangement of body parts between M1 and M2, we first reduced the number of tracked points by focusing on key regions: the head, body and tail. This was done by averaging the spatial coordinates of closely related body parts (e.g., averaging positions of the ears and nose to represent the head). We then calculated the average distances between, e.g., M1 head vs. M2 head, M1 head vs. M2 body, M1 head vs. M2 tail, and all other inter-mouse pairwise body part comparisons. This produced a 3×3 matrix, S, where each cell in S contained the measured distance between the body parts of M1 vs. M2. To identify potential trends in the interaction of M1 and M2, we evaluated the symmetry of S as, $\frac{{\left\|S-S^{T}\;\right\|}_{F}}{{\left\|S\right\|}_{F}}$ , where F denotes the Frobenius norm, which is the Euclidian norm of a matrix with dimensions m x n: ${\left\|S\right\|}_{F}=\sqrt{\sum_{i=1}^{m}\sum_{j=1}^{n}|s_{\mathrm{ij}}|2}$ . Symmetric S-matrices (symmetry ratio ≈ 0) indicate balanced head-body-tail (HBT) interaction, e.g., M1 and M2 maintain similar distances between their heads, bodies, and tails, whereas asymmetric S-matrices (symmetry ratio ≈ 1) indicate disbalance.

### Statistical analysis

To determine significantly different values between experimental conditions, we used a non-parametric bootstrap *t*-test (Efron and Tibshirani, 1993), as previously described (Brockett and Francis, 2024). Given 2 datasets, A and B, having sample sizes of n and m, respectively, we tested A and B against the null hypothesis that they were drawn from a common distribution. The hypothesis test began by taking the absolute value of the observed difference of means, Δμ, between A and B. Next, we created the null distribution by pooling the individual values of A and B. Two sample sets, A* and B*, of size min(n,m), were randomly selected (with replacement) from the null distribution. The test statistic, Δμ*, was computed from the absolute value of the difference of the means obtained from the A* and B* sample sets. We repeated the random selection of A* and B* from the null distribution and the calculation of Δμ*, 10,000 times, to form a bootstrap distribution of Δμ*. A was taken to have a statistically significant different mean than B, if Δμ* was greater than Δμ in less than 5, 1%, or 0.01% of the 10,000 bootstrapped values. This would mean that the probability was <5, 1%, or 0.01% that samples in A and B came from a common distribution. All mean values are reported with standard errors of the mean (SEMs).

### Data and software availability

Data and analysis code are available at: https://bitbucket.org/FrancislabUMD/khan_etal_2025/.

## Results

### BTBR mice show hyperlocomotion

**Figure 2:**
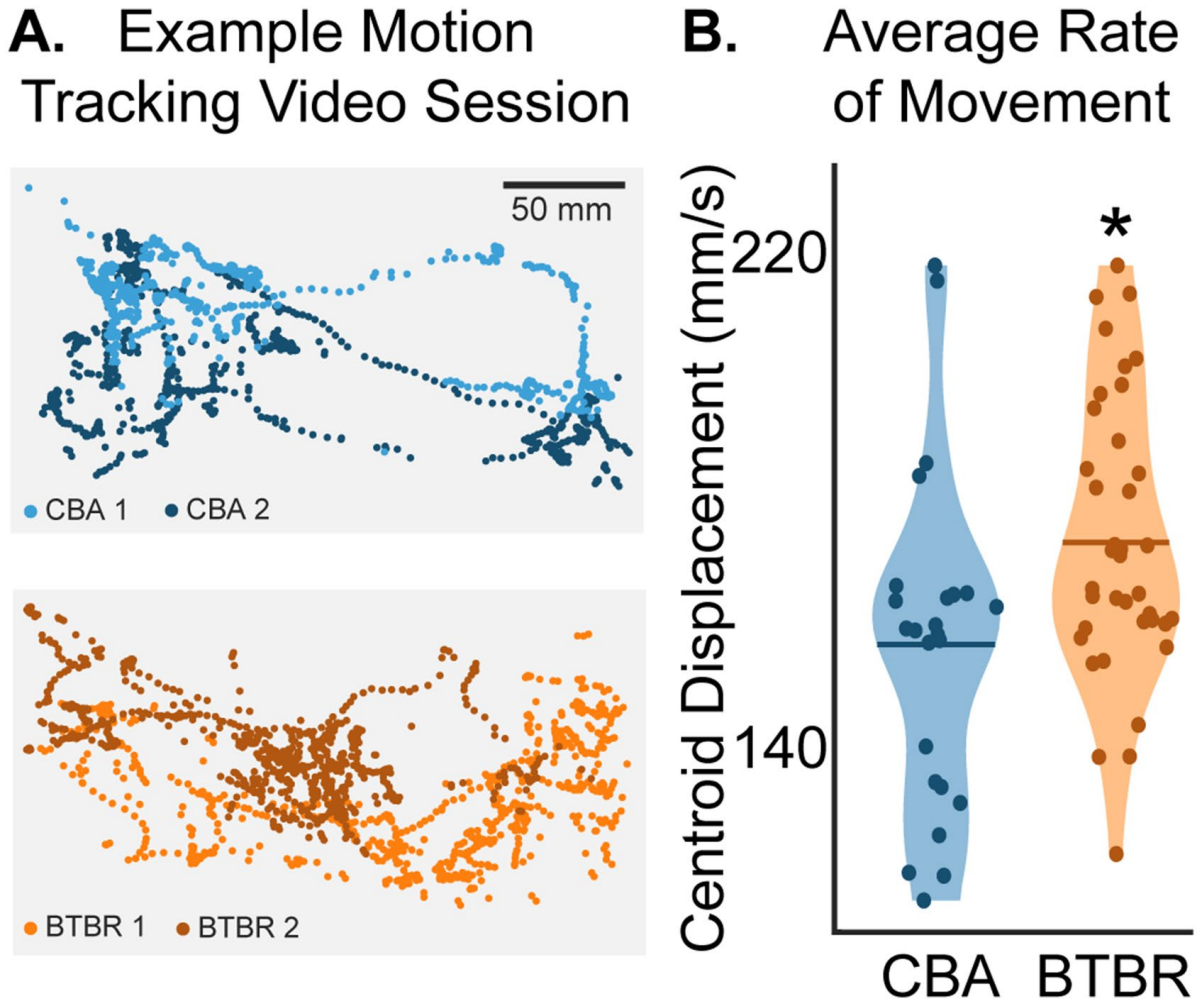
**(A)** Example plots of centroid positions across the 60-min video session. Each mouse is shown using a different color. Each dot (*n* = 900 for both BTBR and CBA mice) shows the frame-by-frame position of a mouse’s centroid across a 30 s epoch. **(B)** Average rate of movement across a video session. The star shows that BTBR centroid displacements were significantly greater than CBA mice (*p* = 0.045, bootstrap t-test with a Bonferroni correction for multiple comparisons of individual mice; *n* = 38 and 24 tracked BTBR and CBA mouse videos, respectively). The horizontal bars show the means of the distributions.

Pairs of familiar mice from the same home cage were placed in a test cage and their behavior over 60 min was scored. We used DeepLabCut (DLC) (Lauer et al., 2022; Mathis et al., 2018) to automatically label frame-by-frame points on body parts from each mouse, including the tail, body, nose, and ears (Figure 1; see Supplementary Video S1). The central position (“centroid”) of each mouse was calculated as the average coordinates of all labeled body parts (white squares in Figure 1B). The trajectory plots in Figure 2A visualize the X-Y positions of the centroids of mice during a session, showing their spatial distribution within the cage across 60 min. To quantify strain differences in locomotion, we computed the average rate of centroid displacement in millimeters per second for each mouse in a video session (Figure 2B). We found that BTBR mice moved more millimeters per second than CBA mice (*p* = 0.045, bootstrap t-test with Bonferroni correction for multiple comparisons). The mean rates of centroid displacements for BTBR and CBA cages were 173 ± 23.0 mm/s and 156 ± 27.0 mm/s, respectively. In other words, BTBR mice tended to move more around the cage, as shown by the larger area covered with dots in the example BTBR and CBA centroid trajectory plots in Figure 2A.

### Familiar BTBR mice exhibit greater social distance than familiar CBA mice

**Figure 3:**
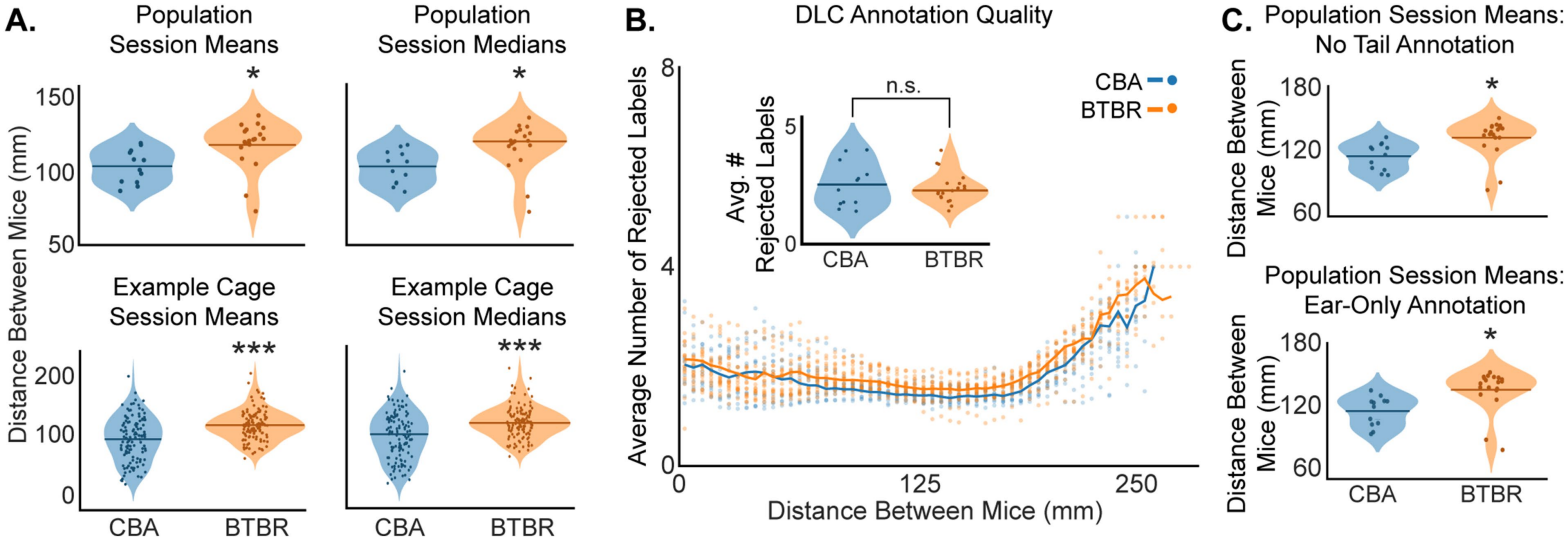
**(A)** BTBR mice show greater social distance than CBA mice. **(A)** Social distance means and medians across all cages (top row, *n* = 19 BTBR and 12 CBA cages), and for example cages (bottom row, *n* = 120 30 s epochs). We found the Euclidean distance between centroids to quantify social distance between pairs of mice in a cage. The stars show that BTBR mice kept a significantly greater distance between mice (top row, mean: *p* = 0.025, median: *p* = 0.035; bottom row, mean: *p* < 0.001, median: *p* < 0.001; bootstrap *t*-test). **(B)** DLC annotation quality was quantified as the average number of rejected body part labels across a video session. Here, it is shown as a function of distance between mice, binned in 5 mm increments. Each dot shows the value from a single video at each of the 120 epochs. The inset shows that the average number of rejected body part labels across all distances was not significantly different for CBA vs. BTBR mice (not significant: n.s.; *p* = 0.64, bootstrap *t*-test). **(C)** The top and bottom panels show that the trend in greater social distance for BTBR vs. CBA mice was robust to both the exclusion of DLC tail annotations (*p* = 0.014, bootstrap t-test) and the exclusive use of ear annotations in calculating centroids (*p* = 0.013, bootstrap *t*-test). *n* = 12 CBA and 19 BTBR cages.

Individual BTBR mice moved more around the cage than CBA control mice (Figure 2B), suggesting that BTBR mice may exhibit more escape behavior. Thus, we sought to understand the relative movement between pairs of familiar mice in a test cage, i.e., social distance. We quantified social distance by finding the mean and median Euclidean distance between centroids of paired mice in sequential 30 s videos within the 60-min video session. This yielded 120 30-s videos per pair of videoed mice. To compare strains, we averaged all 120 centroid distances for each test cage, giving a grand average of both the mean and median social distance per cage. The left columns in Figure 3A show violin plots of the mean and median distances between mice for BTBR and CBA cages. We found that BTBR cages had significantly greater distances between mice (mean: *p* = 0.025; median: *p* = 0.035, bootstrap *t*-test). The mean social distances for CBA and BTBR cages were 103.6 ± 3.20 mm and 117.8 ± 3.62 mm, respectively. The median social distances for CBA and BTBR cages were 104.46 ± 10.93 mm and 121.33 ± 4.25 mm, respectively. In summary, familiar BTBR mice exhibit a greater social distance compared to CBA mice.

### DLC produces reliable estimates of BTBR and CBA position

During DLC annotation, low-confidence predictions of body part labels were removed on a frame-by-frame basis using a p-cutoff value of 0.6. Thus, not all body parts were used to calculate a mouse’s centroid in each frame, which had the potential to bias social distance values, particularly during social interactions at a close distance between mice. Moreover, the difference in fur color for BTBR vs. CBA mice (see Figure 1B) might also have affected the quality of DLC annotation. Hence, in order to rule out the possibility of tracking biases, we assessed the quality of DLC annotation by finding the average number of rejected body part labels as a function of distance between mice, separately for CBA and BTBR mice (Figure 3B). We found very similar patterns of rejections for both mouse strains, wherein approximately 2 labels were rejected for 0–200 mm social distances, which then smoothly increased to approximately 4 rejected labels for 200–250 mm social distances. Importantly, we found no significant difference (*p* = 0.64, bootstrap t-test) in the average number of rejected labels across a given video session for BTBR (2.59 ± 0.28) vs. CBA (2.72 ± 0.52) mice.

We also quantified the percentage of frames in which a label was rejected for each body part, separately for CBA and BTBR mice, across each video session (Table 1). We found that the nose had the highest rejection rate, followed by the tail base, tail end, and was lowest for the ears. This pattern of rejection is most likely explained by behaviors that occlude a body part, such as digging, grooming, or tail tucking. However, we found that calculating social distances after either removing tail labels or after using only the ears gave very similar results as using all points (compare Figures 3A,C). Thus, we found that a DLC p-cutoff value of 0.6 yielded a robust metric of mouse position and continued using all labeled body parts in subsequent analyses.

**Table 1:** Percentage of frames across a video session with a rejected body part (mean ± 2 SEM).

Mouse strain	Tail base	Tail end	Left ear	Right ear	Nose
CBA	34.5% ± 1.7%	26.1% ± 1.3%	12.4% ± 0.9%	12.3% ± 0.9%	42.5% ± 0.9%
BTBR	30.6% ± 1.3%	27.0% ± 1.0%	10.1% ± 0.6%	9.7% ± 0.6%	46.3% ± 0.8%

### Familiar BTBR mice maintain a greater social distance than familiar CBA mice throughout all the time bins of the video session

**Figure 4:**
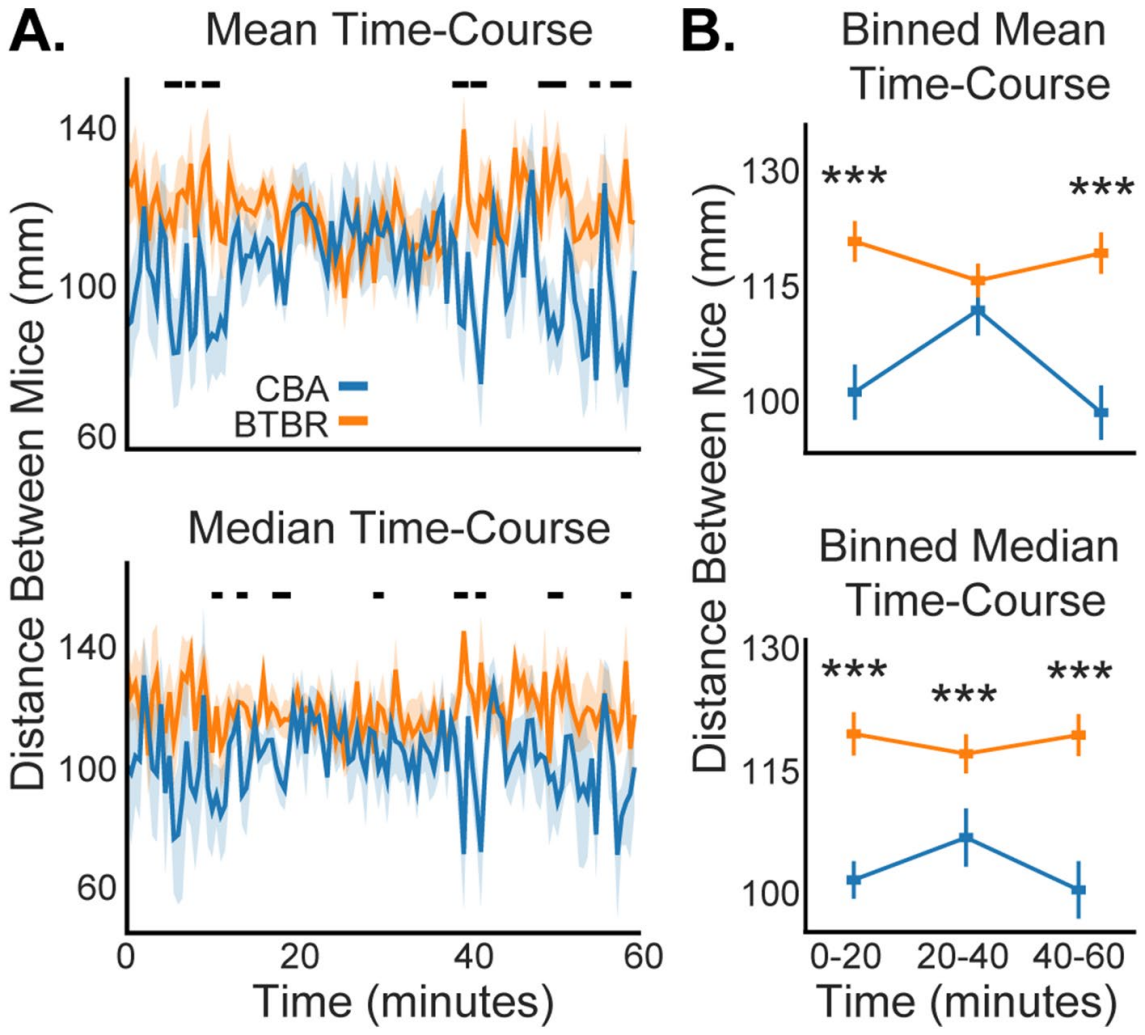
Social distance dynamics across a 60-min video session. **(A)** Familiar BTBR mice show greater social distance than familiar CBA mice throughout an experiment. Shading shows 1 standard error of the mean (SEM). Black dots at the top of the plots show when significant differences occurred between BTBR vs. CBA mice (*p* < 0.05, bootstrap *t*-test). **(B)** We segmented the 60-min video session into three 20-min epochs to clarify the temporal stability of social distance. Stars show when BTBR mice had significantly greater distance between mice during an epoch (see Results for *p*-values, bootstrap *t*-test). *n* = 12 CBA and 19 BTBR cages.

Our use of DLC allowed us to track social distance every 30 s across each 60-min video session, which is plotted in Figure 4A as the mean and median distance between mice, averaged across the populations of n = 19 BTBR and 12 CBA cages. For both the mean and median values, BTBR mice had consistently greater distances between mice across the 60-min session. To clarify trends in the somewhat noisy fine-time plots (Figure 4A), we grouped the centroid data into 3 sequential 20-min time bins and compared social distances for BTBR vs. CBA mice in each time bin (Figure 4B). We found significantly greater social distance for BTBR mice in all median distance time bins (*p* < 0.001, bootstrap *t*-test). While the mean distance values were greater for BTBR mice across all 3 time bins, only the first and last time bins showed a significant difference compared to CBA mice (0–20 min: p < 0.001, 20–40 min: *p* = 0.069, and 40–60 min: p < 0.001).

### Social distance depends on mouse age, sex, and size

**Figure 5:**
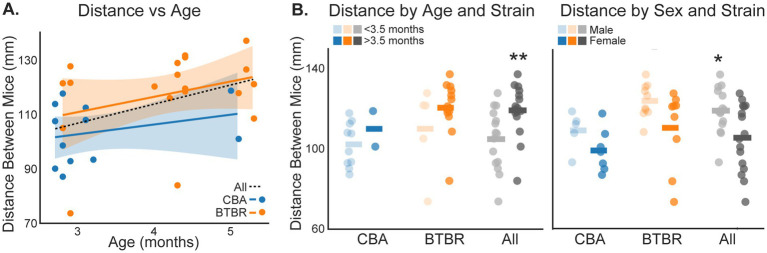
Social distance depends on mouse age and sex. **(A)** Distance between mice plotted against the age of paired mice in each video session. The lines show linear fits to each dataset. The shading shows 2 SEMs. **(B)** The distance between mice for BTBR and CBA groups, and across all mice, was compared for mice <3.5 vs. > 3.5 months old (left panel), and for males vs. females (right panel). Horizontal bars show group means. Stars show significant differences for the All-mice group: <3.5 vs. > 3.5 month old mice (*p* = 0.009, bootstrap t-test) and males vs. females (*p* = 0.014, bootstrap *t*-test). *n* = 5 male and 7 female CBA mice. *n* = 10 male and 9 female BTBR mice. *n* = 10 < 3.5 and 2 > 3.5 month old CBA mice. *n* = 5 < 3.5 and 14 > 3.5 month old BTBR mice.

Within our BTBR and CBA populations, the mice varied in age, sex, and size. Thus, we sought to understand how these factors might influence social distance. Paired mice in each video were the same age, since they were littermates. Figure 5A shows the distance between mice plotted against the age of the mice in each video. While there was a trend towards increased distance between mice as age increased, we did not find a significant correlation within BTBR (r = 0.31, *p* = 0.204) or CBA (r = 0. 257, *p* = 0.421) groups. However, when considering all mice grouped together, there was a significant correlation (r = 0.44, *p* = 0.014). Since most CBAs were less than 3.5 months old, and most BTBRs were more than 3.5 months old, we grouped mice within each strain and across all mice into <3.5 or >3.5 months old and compared social distance in each group (Figure 5B). As in the correlation analysis, we did not find a significant difference in the distance between mice for <3.5 vs. > 3.5 months old mice within the BTBR or CBA groups (*p* > 0.05, bootstrap t-test). However, age had a significant effect after grouping both strains (*p* = 0.009, bootstrap t-test). The mean social distances for all <3.5 and >3.5 month old cages were, 104.92 ± 7.84 mm and 119.28 ± 6.46 mm, respectively.

BTBR mice exhibit sex-specific ASD-like behaviors (Defensor et al., 2011; Bove et al., 2024; Amodeo et al., 2019). Thus, we compared social distance in males vs. females, for CBA, BTBR, and across all mice. While the distance between mice tended to be greater for males vs. females, we did not find a sex-based significant difference within BTBR or CBA groups (*p* > 0.05, bootstrap t-test). However, sex had a significant effect after grouping both strains (*p* = 0.014, bootstrap *t*-test). The mean social distances for all males and females were, 119.29 ± 5.77 mm and 105.82 ± 8.34 mm, respectively.

**Figure 6:**
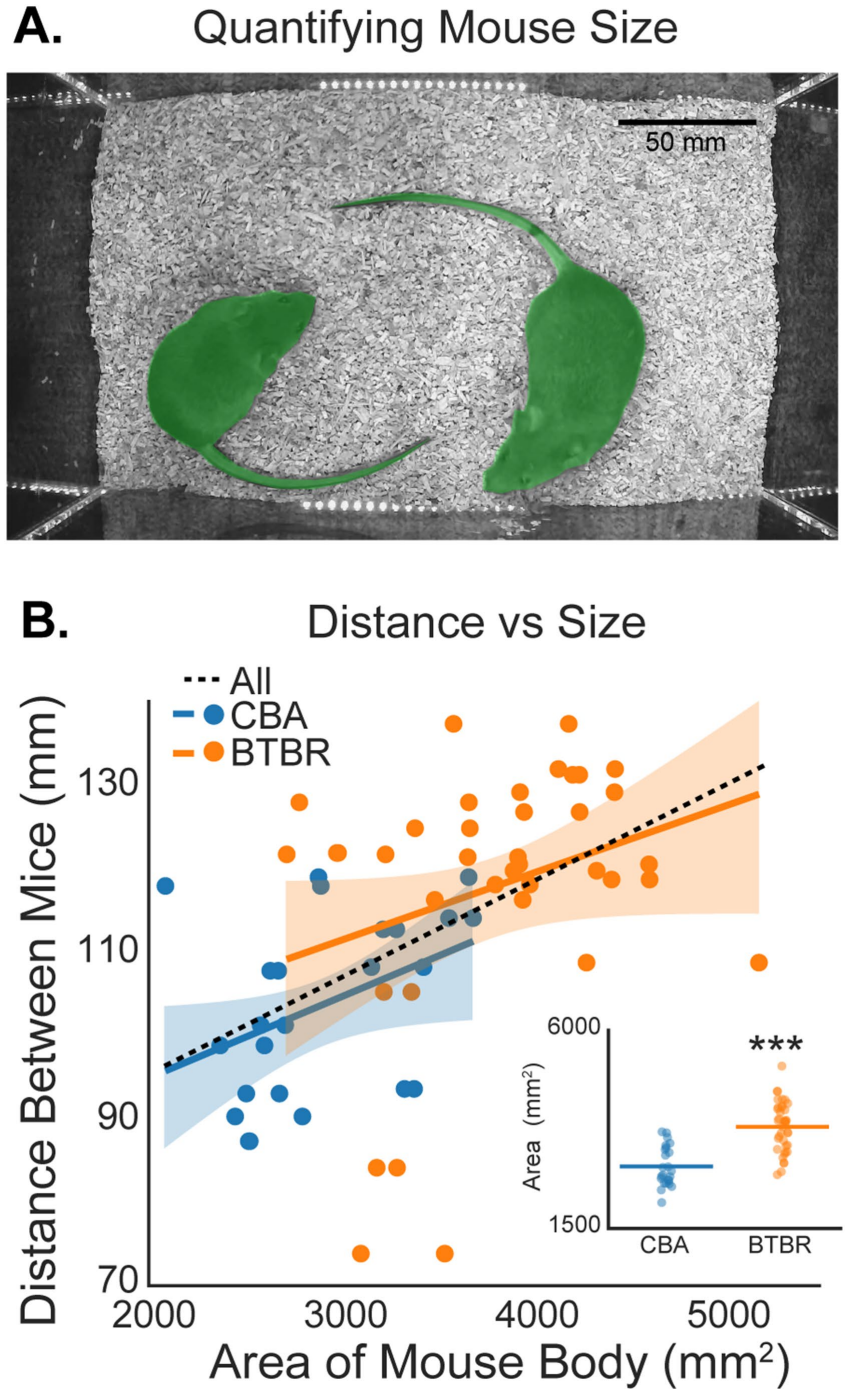
Social distance depends on mouse size. **(A)** Example of polygon masks (green overlaid on each mouse) used to estimate the size of each mouse in a video session. Masks were used to estimate the area (mm^2^) of each mouse. **(B)** The distance between mice plotted against the area of each mouse. Stars in the inset show that BTBR mice were significantly larger than CBA mice (*p* < 0.001, bootstrap *t*-test). *n* = 12 CBA and 19 BTBR cages.

Body size is another factor that may influence social distance, since bigger mice occupy a larger area within the video arena. We quantified mouse size by finding the area (mm^2^) occupied by each mouse in the video field of view (see Methods; Figure 6A). The inset in Figure 6B shows that BTBR mice were significantly larger than CBA mice (*p* < 0.001, bootstrap t-test). The mean areas for CBA and BTBR mice were, 2904.54 ± 184.14 mm^2^ and 3807.87 ± 183.30 mm^2^, respectively. Figure 6B also shows the distance between mice plotted against the size of each mouse in our experiment. While there was a trend towards increased distance between mice as size increased, we did not find a significant correlation within BTBR (*r* = 0.29, *p* = 0.076) or CBA (*r* = 0.402, *p* = 0.052) mice. However, when considering all mice grouped together, there was a significant correlation (*r* = 0.51, *p* < 0.001). Together, our data suggest that social distance depends on mouse age, sex, and size.

### BTBR and CBA mice have similarly balanced head-body-tail interactions between cagemates

**Figure 7:**
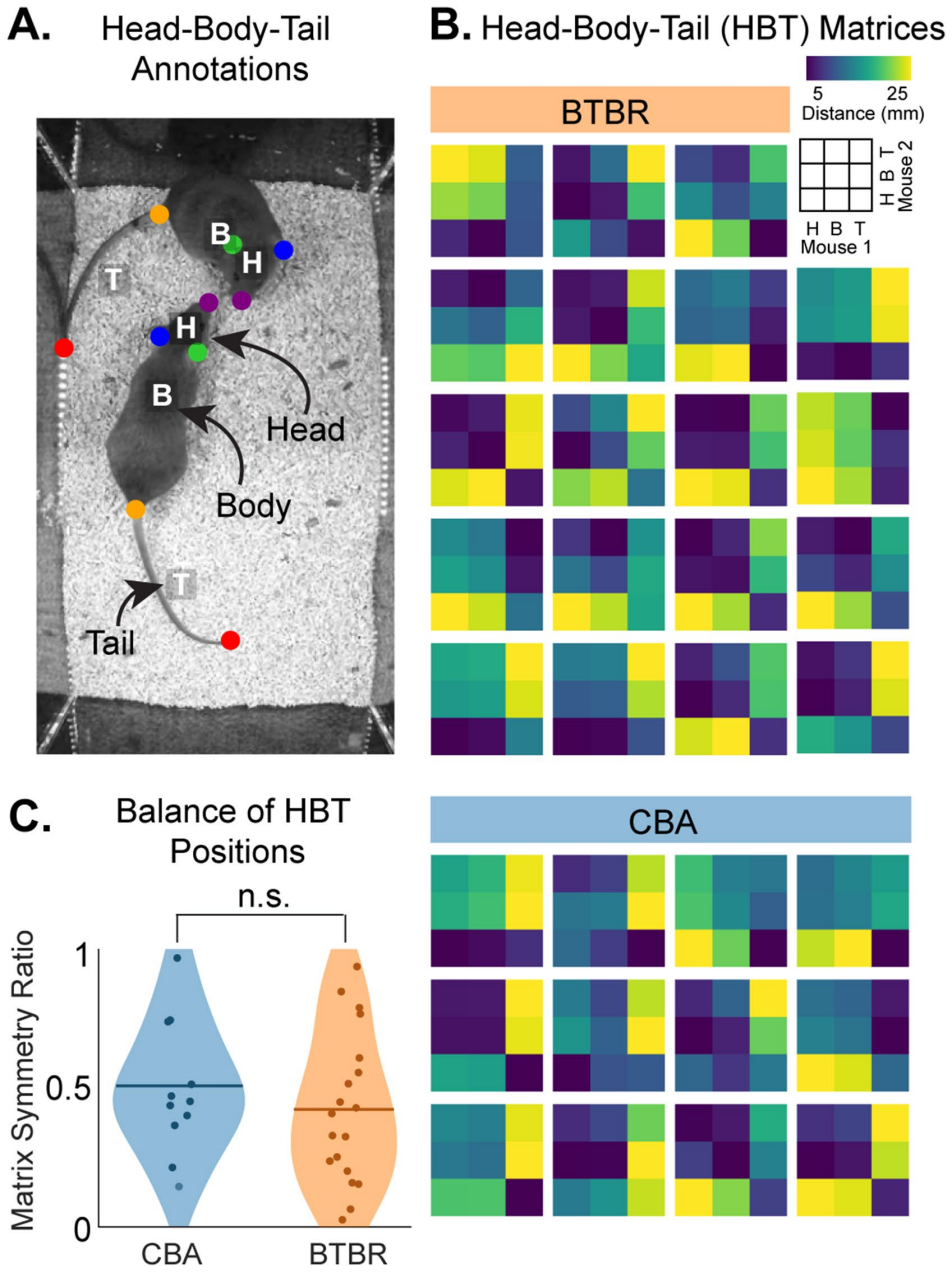
BTBR and CBA mice show similarly balanced social interactions. **(A)** We quantified socio-spatial arrangements of mice using a reduced set of body parts in each mouse: Head (H), Body (B), and (Tail) (HBT). **(B)** HBT distance matrices. We measured the pairwise distances for H, (B), and T between the pair of mice in a cage (see color bar). The legend shows the HBT matrix pairwise comparisons. **(C)** The balance of socio-spatial arrangements between pairs of mice was quantified by finding the symmetry of each HBT matrix (see Methods). A symmetry ratio closer to 1 indicates greater asymmetry, i.e., disbalanced social interaction. 0 indicates perfect symmetry, i.e., balanced social interaction. We found similar symmetry ratios for both CBA and BTBR mice (*p* = 0.4, bootstrap t-test, *n* = 12 CBA and 19 BTBR cages).

So far, we have evaluated distance metrics between the centroids from pairs of mice. However, social interactions in mice, such as allogrooming, sniffing, and huddling, involve relative proximities of different body parts. For example, during sniffing, the head of mouse 1 might be near the head of mouse 2, while their tails are far apart. To analyze social interaction in mice at a finer spatial scale, we tracked 3 key regions on each mouse: the head, body and tail (Figure 7A) (see Methods). We then calculated the average distances between, e.g., M1 head vs. M2 head, M1 head vs. M2 body, M1 head vs. M2 tail, and all other inter-mouse pairwise body part comparisons. This produced a 3×3 Head-Body-Tail (HBT) matrix, S, where each cell in S contained the measured distance between the body parts of M1 vs. M2. Figure 7B shows the HBT matrix, ‘S’, for each cage.

The variety of HBT matrices shown in Figure 7B indicates the diversity of social behaviors in mice. To identify potential trends in the interaction of M1 and M2, we evaluated the symmetry of S (see Methods). Symmetric, ‘S’, matrices (symmetry ratio ≈ 0) indicate balanced HBT interaction, e.g., M1 and M2 maintain similar distances between their heads, bodies, and tails, whereas asymmetric matrices (symmetry ratio ≈ 1) indicate disbalance, e.g., M1 consistently positioned its head closer to M2’s tail. Figure 7C shows violin plots of the symmetry ratios for BTBR and CBA cages. The average symmetry ratio values were 0.51 and 0.43 for BTBR and CBA cages, respectively, though there was no significant difference (*p* = 0.4, bootstrap t-test). Thus, we found similarly balanced HBT interactions within BTBR and CBA cages.

## Discussion

Using DeepLabCut (DLC) to analyze video of mouse behavior, we found that familiar pairs of BTBR T^+^ Itpr3^tf^/J (BTBR) mice show significantly greater social distances compared to CBA control mice, consistent with BTBR social avoidance of stranger mice (Choi et al., 2020; Teo et al., 2013; Lamu and Olsen, 2016; McFarlane et al., 2008; Meyza and Blanchard, 2017; Ellegood et al., 2013; Scattoni et al., 2013). Our use of DLC allowed us to quantify metric units of social distance, showing that BTBR mice kept on average an additional 14.2 mm between individuals. Moreover, by recording social behavior for 60 min, we showed that increased BTBR social distancing is not transient, but rather it is maintained throughout the testing period. We also confirmed previous reports that BTBR mice exhibit hyperlocomotion (Amodeo et al., 2019; Shrader et al., 2024). Our results emphasize the translational value of BTBR mice in understanding ASD-related social behavior. While many of the behaviors previously observed in BTBR mice have been evaluated and scored manually by experimenters, here we demonstrate the usefulness of DLC for automated analysis of BTBR behavior.

We found that several phenotypic factors including age, sex, and body size may contribute to social distancing in mice. Thus, future experiments will need to control for these phenotypes before interpreting social *distance* as social *avoidance* in BTBR mice. Yet, it is important to note that sex-based differences in ASD-related social behavior occur in humans and are mirrored in BTBR mice. Social masking that obscures autistic traits is more prevalent among human females than males (Green et al., 2019; Hull et al., 2020; Simcoe et al., 2023; McQuaid et al., 2022). Human females with autism also tend to have greater social motivation and increased sensitivity to social expectations (Green et al., 2019; Simcoe et al., 2023). In BTBR mice, males show low sociability in general, while females demonstrate more variable social engagement depending on the social context (Defensor et al., 2011; Ryan et al., 2019). We did not find significant effects of sex on social distance within the BTBR or CBA mouse groups, but males did show significantly greater social distance when all mice were grouped together, and trends within both strains followed trends in the overall population. In a similar manner, we found that social distance increased as mice aged, highlighting a potential role for BTBR mice in studying the age-related dynamics of social behavior.

Hong et al. (2015) also analyzed BTBR social behavior but observed less pronounced social distancing than we found in our experiments. The variable results across studies highlight the complexity of assessing social interactions between mice in different experimental setups. While Hong et al. (2015) used depth-sensing technology to measure social distance, our experiment used 2-dimensional images from a typical digital camera. In addition, we allowed 60 min for mouse interactions, whereas Hong et al. (2015) only allowed 15 min and the familiarity of the paired mice in their study was unclear. These methodological differences may explain our contrasting results regarding social distancing.

Finally, we found that BTBR mice displayed similarly balanced head-body-tail (HBT) interactions compared to the CBA controls, whereas previous work reported that BTBR mice avoided face-to-face and face-to-body interactions (Ellegood et al., 2013; Scattoni et al., 2013; Yang et al., 2012; Defensor et al., 2011). The differing results might arise from our quantification of social interaction based on aggregate body-part distances. However, a primary difference in our study was the pairing of familiar mice, rather than the pairing of stranger mice. Thus, social familiarity may modulate how BTBR mice use face-to-face and face-to-body interactions. In summary, we find that DLC facilitates accurate quantification of social behavior in BTBR mice. More generally, DLC-mediated quantification of social distance could in the future become a valuable tool for social research with mice as well as with other species.

## Data Availability

The datasets presented in this study can be found in online repositories. The names of the repository/repositories and accession number(s) can be found below: https://bitbucket.org/FrancislabUMD/khan_etal_2025/.
