# Supplementary material for: Quantifying social distance using deep learning-based video analysis: results from the BTBR mouse model of autism
Source: Front Behav Neurosci. 2025 Jun 20;19:1602205. doi: 10.3389/fnbeh.2025.1602205 (PMC12226490; doi:10.3389/fnbeh.2025.1602205)
Supplement: Supplementary file 1 [file Data_Sheet_1.pdf]

Supplemental Information for, "Quantifying Social Distance Using Deep Learning-Based Video Analysis: Results from the BTBR Mouse Model of Autism"

TITLE

Quantifying Social Distance Using Deep Learning-Based Video Analysis: Results from the BTBR Mouse Model of Autism

AUTHORS

Tausif Khan[1,2], Kostiantyn Cherkas[3], and Nikolas A. Francis[1,3,*]

AUTHOR AFFILIATIONS

[1]Department of Biology, University of Maryland, College Park, MD, 20742

[2]Program in Applied Machine Learning, University of Maryland, College Park, MD, 20742

[3]Brain and Behavior Institute, University of Maryland, College Park, MD, 20742

*Corresponding author

CORRESPONDING AUTHOR

Nikolas A. Francis (cortex@umd.edu)

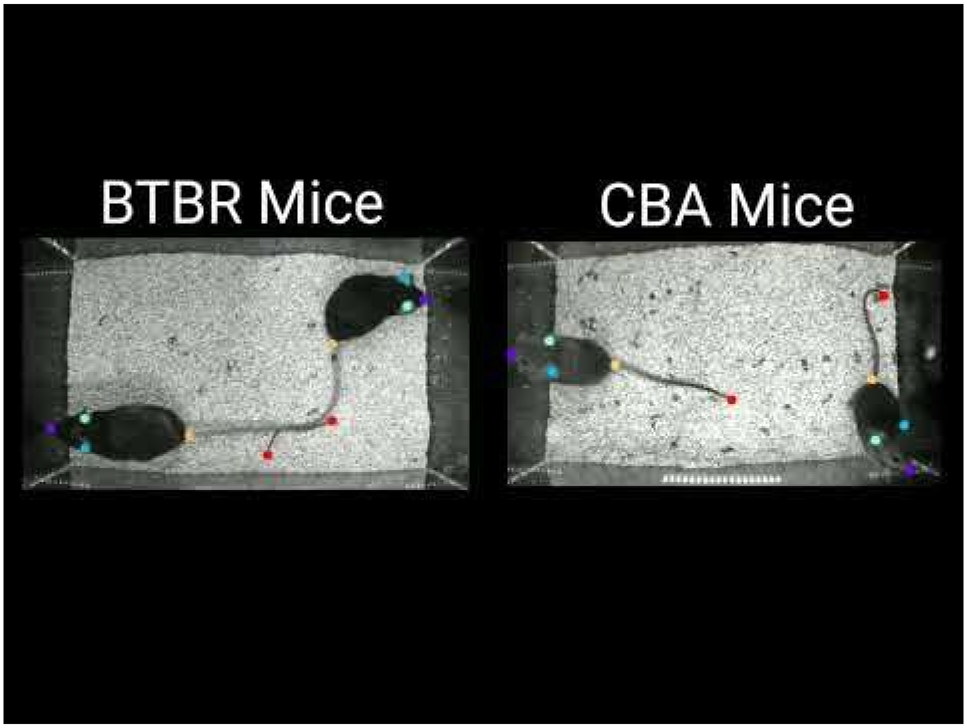

Supplementary Video S1 (click the image to access video). Video labeled by DeepLabCut. We used DeepLabCut to generate labeled key points on each mouse (*see* Methods). Colored dots show accurate frame-by-frame labeling of key points on each mouse.

Comparative Neuroanatomy: Corpus Callosum (CC) and Anterior Commisure (AC) in CBA versus BTBR mice

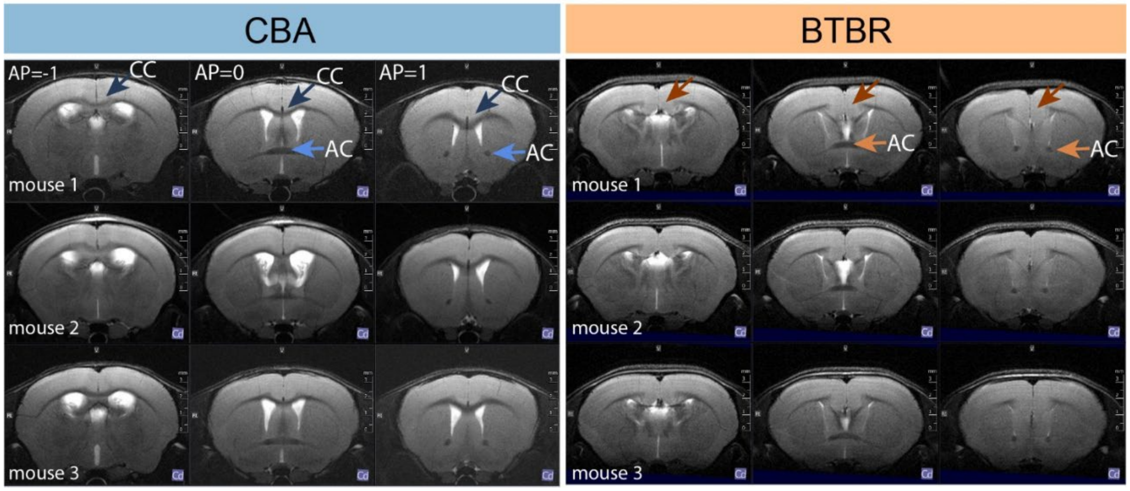

Supplementary Figure S1. Our study aimed to assess social distance in BTBR T^{+} $Itpr3^{tf}$/J ("BTBR") mice, a strain often used to investigate the physiological basis of Autism Spectrum Disorder (ASD). Corpus callosum (CC) agenesis is a neuroanatomical phenotype observed in BTBR mice (1-5), and CC dysgenesis is thought to be more frequent in individuals with ASD (6-9). We verified CC agenesis in three BTBR mice using magnetic resonance imaging (MRI). For both CBAs and BTBRs, each row of images shows a different mouse, and each column shows a coronal slice from just posterior to the anterior-posterior (AP) midline (AP=-1), at the AP midline (AP=0), and just anterior to the AP midline (AP=1). The images show how both strains have similar neuroanatomy for the Anterior Commissure (AC), but there was no CC in BTBR mice.

MRI experiments (see supplementary figure S1) were performed on a horizontal bore Bruker BioSpec 94/30 USR MR system (9.4 Tesla, Billerica, MA, USA) with an actively shielded 12 cm diameter insert, a maximum gradient strength of 440 mT/m, and a rise time of 130 μs. A quadrature birdcage coil (86 mm inner diameter) was used for excitation, while 2 x 2 array cryogenically-cooled RF coil positioned on top of the mouse's head was used for detection. High resolution T1-weighted anatomical images were obtained in the sagittal plane using fast low angle shot MRI sequence (FLASH) with the following parameters: TR/TE = 560/6 ms, NEX = 40, and flip angle (FA) = 35°, field of view = 20 (readout) × 20 (phase encoding) mm2, matrix = 400 × 400, in-plane resolution = 50 μm × 50 μm, slice thickness = 500 μm, fat suppression with a bandwidth of 1.4 kHz (pulse shape "gauss512", duration ~ 2.75 ms) and contiguous 9 slices without gap. Total scan time was ~2 hours. All MRI experiments were performed under 2% isoflurane anesthesia. Body temperature was maintained at 37 ± 0.5 °C through the rectal temperature probe. Electrocardiogram (ECG) and motion-sensitive respiration signals were continuously monitored (Small Animal Instruments Inc; Acknowledge, Biopac Systems, Inc., Goleta, CA, USA). Imaging datasets were converted to NIfTI format and organized into the Brain Imaging Data Structure (BIDS) (10). Data analyses were performed with the Analysis of Functional Neuroimages package (AFNI) (11), FMRIB Software Library (FSL) (12), and Advanced Normalization Tools (ANTs) (13) within the custom-built data processing pipeline made with Nipype (14). Low frequency intensity non-uniformity was normalized using the N4 algorithm (15).

Supplementary References

1. Morcom L, Edwards TJ, Rider E, Jones-Davis D, Lim JW, Chen KS, et al. DRAXIN regulates interhemispheric fissure remodelling to influence the extent of corpus callosum formation. Elife. 2021;10.
2. Dodero L, Damiano M, Galbusera A, Bifone A, Tsaftsaris SA, Scattoni ML, et al. Neuroimaging evidence of major morpho-anatomical and functional abnormalities in the BTBR T+TF/J mouse model of autism. PLoS One. 2013;8(10):e76655.
3. Fenlon LR, Liu S, Gobius I, Kurniawan ND, Murphy S, Moldrich RX, et al. Formation of functional areas in the cerebral cortex is disrupted in a mouse model of autism spectrum disorder. Neural Dev. 2015;10:10.
4. Martin LA, Hsu FW, Herd B, Gregg M, Sample H, Kaplan J. Executive functions in agenesis of the corpus callosum: Working memory and sustained attention in the BTBR inbred mouse strain. Brain Behav. 2021;11(1):e01933.
5. Miller VM, Gupta D, Neu N, Cotroneo A, Boulay CB, Seegal RF. Novel inter-hemispheric white matter connectivity in the BTBR mouse model of autism. Brain Res. 2013;1513:26-33.
6. Alexander AL, Lee JE, Lazar M, Boudos R, DuBray MB, Oakes TR, et al. Diffusion tensor imaging of the corpus callosum in Autism. Neuroimage. 2007;34(1):61-73.
7. Frazier TW, Keshavan MS, Minshew NJ, Hardan AY. A two-year longitudinal MRI study of the corpus callosum in autism. J Autism Dev Disord. 2012;42(11):2312-22.
8. Frazier TW, Hardan AY. A meta-analysis of the corpus callosum in autism. Biol Psychiatry. 2009;66(10):935-41.
9. Travers BG, Tromp do PM, Adluru N, Lange N, Destiche D, Ennis C, et al. Atypical development of white matter microstructure of the corpus callosum in males with autism: a longitudinal investigation. Mol Autism. 2015;6:15.
10. Gorgolewski KJ, Auer T, Calhoun VD, Craddock RC, Das S, Duff EP, et al. The brain imaging data structure, a format for organizing and describing outputs of neuroimaging experiments. Sci Data. 2016;3:160044.
11. Cox RW. AFNI: software for analysis and visualization of functional magnetic resonance neuroimages. Comput Biomed Res. 1996;29(3):162-73.
12. Smith SM, Jenkinson M, Woolrich MW, Beckmann CF, Behrens TE, Johansen-Berg H, et al. Advances in functional and structural MR image analysis and implementation as FSL. Neuroimage. 2004;23 Suppl 1:S208-19.
13. Avants BB, Tustison NJ, Song G, Cook PA, Klein A, Gee JC. A reproducible evaluation of ANTs similarity metric performance in brain image registration. Neuroimage. 2011;54(3):2033-44.
14. Gorgolewski K, Burns CD, Madison C, Clark D, Halchenko YO, Waskom ML, et al. Nipype: a flexible, lightweight and extensible neuroimaging data processing framework in python. Front Neuroinform. 2011;5:13.
15. Tustison NJ, Avants BB, Cook PA, Zheng Y, Egan A, Yushkevich PA, et al. N4ITK: improved N3 bias correction. IEEE Trans Med Imaging. 2010;29(6):1310-20.
